# Circulating Mitochondrial DNA Aggravates Post-Ischemic Functional and Metabolic Recovery in an Isolated Rat Heart Model of Donation After Circulatory Death

**DOI:** 10.3390/ijms27104360

**Published:** 2026-05-14

**Authors:** Maria Nieves Sanz, Maria Arnold, Adrian Segiser, Michelle Hofmann, Matthias Siepe, Sarah L. Longnus

**Affiliations:** 1 Department of Cardiac Surgery, Inselspital, Bern University Hospital, University of Bern, 3010 Bern, Switzerland; 2 Department for BioMedical Research, University of Bern, 3008 Bern, Switzerland

**Keywords:** donation after circulatory death, ischemia–reperfusion injury, heart transplantation, mitochondrial damage-associated molecular patterns

## Abstract

During donation after circulatory death (DCD), circulating levels of mitochondrial damage-associated molecular patterns (mtDAMPs) may increase, thereby exposing donor hearts to mtDAMPs prior to procurement and during machine perfusion. Mitochondrial DNA (mtDNA) is a pro-inflammatory mtDAMP that may stimulate several intracellular cascades including that of toll-like receptor 9 (TLR9). We administered mtDNA or ODN2088 (TLR9 antagonist) to hearts at reperfusion onset using an isolated rat heart model of DCD transplantation to investigate their effects. Four experimental groups were compared: (1) no ischemia; (2) ischemia; (3) ischemia + mtDNA; (4) ischemia + ODN2088. During reperfusion, cardiac power in ischemic hearts was significantly reduced compared to non-ischemic hearts (*p* < 0.01), and was further decreased with mtDNA (*p* < 0.05), but remained unchanged with ODN2088. Reduced ventricular recovery in mtDNA-treated hearts likely resulted from lower recovery of oxidative metabolism, demonstrated by reduced oxygen efficiency (*p* < 0.05) and a strong tendency for increased cytochrome c release (*p* < 0.06),indicating mitochondrial dysfunction and disruption, respectively. ODN2088 phosphorylated IκBα (NF-κB inhibitor alpha) and appeared to decrease cardiomyocyte death compared to ischemic hearts. Given the detrimental effects of circulating mtDNA on cardiac functional and metabolic recovery, circulating mtDAMPs, and particularly mtDNA, are of clinical relevance as potential therapeutic targets for optimizing graft quality and post-transplant outcomes.

## 1. Introduction

Heart failure is a progressive disease, affecting more than 64 million people globally [1]. For patients with advanced heart failure, heart transplantation remains the gold-standard therapy to improve quality of life and survival. However, insufficient supply of grafts currently limits the number of heart transplantations. One promising solution for increasing cardiac graft availability is the use of hearts obtained with donation after circulatory death (DCD). Indeed, DCD heart transplantation has provided excellent five-year and eight-year outcomes [2,3,4] as well as improvements in heart transplantation rates by 25–48% [4,5,6], demonstrating both the feasibility and benefits of this approach.

However, DCD heart transplantation still raises concern as, unlike conventional donation after brain death (DBD) organs, DCD hearts undergo a period of global, warm ischemia prior to procurement. This critical period, in which application of cardioprotective measures are not permitted for ethical and legal reasons, can lead to irreversible damage of DCD grafts [7,8]. Paradoxically, reperfusion, which is required to salvage cardiac cell viability, triggers tissue damage termed ischemia–reperfusion (IR) injury. IR injury is a critical factor in functional and survival outcomes of high-energy demand organs, such as cardiac tissue. Thus, a more comprehensive understanding of metabolic mediators triggered by IR injury in DCD hearts during the first moments of reperfusion, when application of cardioprotective strategies that can lessen this damage are permitted, is key in optimizing tolerance of DCD hearts to IR injury and positively impacting further organ performance.

Mitochondria play a central role in the metabolic performance of cardiac tissue and are very sensitive organelles to IR injury. Mitochondria damaged by IR injury release a specific class of damage-associated molecular patterns (DAMPs), called mitochondrial DAMPs (mtDAMPs). Recognized mtDAMPs include mitochondrial DNA (mtDNA), cytochrome c (Cyt c), ATP, mitochondrial transcription factor A, N-formyl peptides, succinate and cardiolipin. mtDAMPs are able to initiate innate inflammatory responses [9], most likely due to their bacterial origin [10]. Of all mtDAMPs, mtDNA has been reported as the most pro-inflammatory [11]. mtDNA is a double-stranded circular molecule with thousands of copies per cell, contains unmethylated CpG motifs and is recognized by toll-like receptor 9 (TLR9), mimicking bacterial DNA, and other cellular triggers of innate inflammation such as the NLRP3 inflammasome and cyclic GMP-AMP synthetase (cGAS)-STING (stimulator of interferon gene) DNA-sensing pathways [12]. It also lacks the packaging of histones, rendering it more susceptible to oxidation by mitochondrial ROS produced in the vicinity.

Strikingly, and despite the potential of mtDNA as a propagator of the inflammation associated with IR injury, little is known about its role in DCD-induced injury in the heart. In a swine model of kidney donation, longer periods of normothermic reperfusion, a technique used to preserve procured organs prior to transplantation, were associated with a continuous release of mtDNA into the perfusate [13]. Furthermore, in human liver transplantation, higher levels of circulating mtDNA at organ procurement have been reported in both DBD and DCD donors compared to living donors [14]. Interestingly, a correlation between donor plasma mtDNA levels and early allograft dysfunction in liver transplant recipients was also reported [14]. This and other studies have shown that circulating mtDNA levels measured in human DCD donors prior to organ procurement and/or during further graft reperfusion might be good candidates to predict early graft dysfunction in recipients of DCD liver [14], kidney [15] or lung [16] grafts, and are associated with poorer post-ischemic function. In the context of DCD heart transplantation, cardiac grafts are likely exposed to elevated levels of circulating mtDNA in the donor as a result of periods of hypoxia, hemodynamic instability and ischemia prior to procurement. Furthermore, during machine perfusion, grafts may initially be exposed to mtDNA present in the donor blood used in the perfusate, but are then likely subjected to much higher levels following reperfusion and mtDAMP release by the heart into a relatively small perfusate volume without specific clearance mechanisms.

Among mtDNA-mediated innate inflammation pathways, modulation of TLR9 activity has emerged as a promising therapy for protection against IR injury. TLR9 can act through two mechanisms of action: (1) by triggering a pro-inflammatory response [17]; or (2) by reducing mitochondrial ATP production through the inhibition of SERCA2 [18]. In a porcine model of liver DCD, the TLR9 antagonist (ODN2088) preserved hepatic tissue from warm ischemia-induced damage at histological and functional (release of aminotransferase) levels [19]. The treatment with ODN2088 significantly reduced protein content of TLR9, as well as inflammatory regulators interferon regulatory factor 7 (IRF7), tumor necrosis factor α (TNF-α) and interferon β (IFN-β) in hepatic tissue harvested after global ischemia [19]. Consistent with these findings, pre-ischemic treatment with ODN2088 reduced myocardial infarct size by inhibiting the TLR9-type I interferon pathways in a mouse model [20]. However, ODN2088 has also provided protective effects in mice through the activation of phosphoinositide 3 kinase (PI3K)/Akt signaling to reduce cerebral IR injury, and through PI3K/Akt and extracellular-signal-related kinase signaling to prevent sepsis-induced cardiac dysfunction [21]. Despite these promising findings of ODN2088 as a potential protective therapy, it is important to note that the approaches used in these studies are not applicable to clinical DCD heart transplantation, as ODN2088 was administered prior to ischemia (or sepsis) onset, and interventions to protect DCD organs are generally not permitted before organ ischemia/donor death.

We propose that mtDNA, present in the donor circulation and/or following release from mitochondria of DCD hearts during machine perfusion, activates TLR9 and leads to the initiation of innate inflammatory responses and a potential decline in cardiac performance. Correspondingly, inhibition of TLR9 during reperfusion of DCD hearts will reduce innate inflammatory responses and may improve cardiac post-ischemic recovery. As a first investigation into this concept, we investigated the effects of intravascular administration of extracellular mtDNA or an antagonist of TLR9 at reperfusion onset after a period of global, warm ischemia using an isolated rat heart model of DCD. The experimental model was chosen to simulate the period of machine perfusion used in the storage/transport of DCD hearts, as this is the moment when cardioprotective strategies can be applied.

## 2. Results

### 2.1. Baseline Characteristics

Several contractile and metabolic parameters, determined at 20 min baseline perfusion, as well as anatomic features, are presented in Table 1. No significant difference among groups was detected for functional variables (left ventricular power, cardiac output or coronary flow), or for body and heart weights. For metabolic parameters, oxygen consumption in ischemic hearts treated with mtDNA was significantly greater than that in ischemic hearts treated with ODN2088 (*p* < 0.01).

### 2.2. Contractile Function During Reperfusion

**Figure 1:**
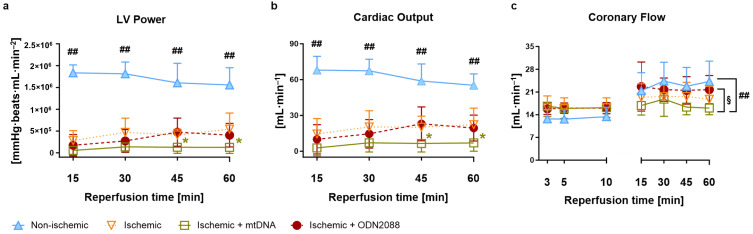
Post-ischemic cardiac function. (**a**) LV power (left ventricular power: heart rate–developed pressure–cardiac output product); (**b**) CO (cardiac output); (**c**) CF (coronary flow). ^##^ *p* < 0.01 vs. all ischemic groups or between indicated groups; * *p* < 0.05 vs. ischemic; ^§^ *p* < 0.05 between indicated groups; min: minutes; *n* = 6 to 12 per group.

Left ventricular (LV) power (Figure 1a), calculated as the product of developed pressure, heart rate, and cardiac output, was significantly lower in all ischemic groups compared to the non-ischemic group at all times of reperfusion monitored (15, 30, 45 and 60 min; *p* < 0.01 for all). Interestingly, intracoronary infusion of mtDNA to ischemic hearts at the beginning of reperfusion significantly impaired LV power at 45 and 60 min reperfusion compared to non-treated ischemic hearts (*p* < 0.05; both timepoints). A similar pattern was found when cardiac output was investigated (Figure 1b). Coronary flow (Figure 1c) was similar during early reperfusion (3, 5 and 10 min) among all groups, indicating that treatments administered to ischemic groups did not interfere with coronary perfusion and remained similar among groups for the rest of reperfusion; however, a tendency towards a lower coronary flow in ischemic hearts treated with mtDNA was observed, reaching statistical significance at the end of reperfusion compared to non-ischemic hearts and hearts treated with ODN2088 (*p* < 0.05 vs. ischemic + ODN2088; *p* < 0.01 vs. non-ischemic).

### 2.3. Cell Death During Reperfusion

**Figure 2:**
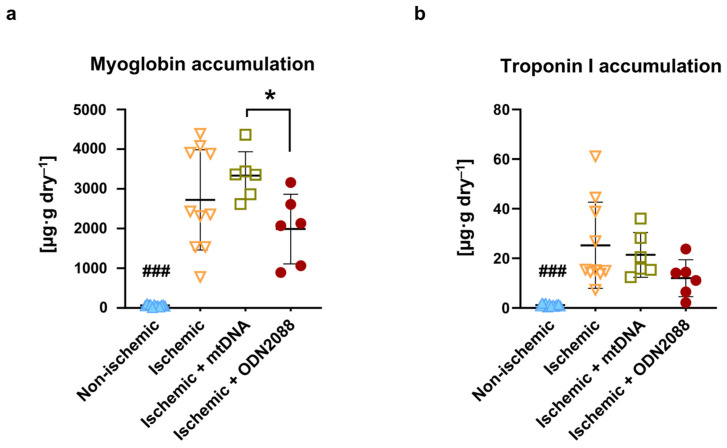
Release of markers of cell death during reperfusion. Content of (**a**) myoglobin and (**b**) TnI accumulated over 60 min reperfusion in perfusate. TnI: troponin I; ^###^ *p* < 0.001 vs. all ischemic groups; * *p* < 0.05 between indicated groups; *n* = 6 to 12 per group.

Myoglobin and cardiac troponin I (TnI) levels, markers of cell death, were quantified in perfusate collected at onset and end of reperfusion, and accumulation over the reperfusion period was subsequently calculated (Figure 2). All ischemic groups showed significantly higher levels of both markers (myoglobin: Figure 2a; TnI: Figure 2b) compared to non-ischemic hearts (*p* < 0.001). Ischemic hearts treated with mtDNA appeared to accumulate the highest level of myoglobin of all groups included in the study, reaching statistical significance when compared with hearts treated with ODN2088 (*p* < 0.05). Ischemic hearts treated with ODN2088 tended to accumulate the lowest levels of markers of cell death compared to the other two ischemic groups.

### 2.4. Metabolic Function During Reperfusion

**Figure 3:**
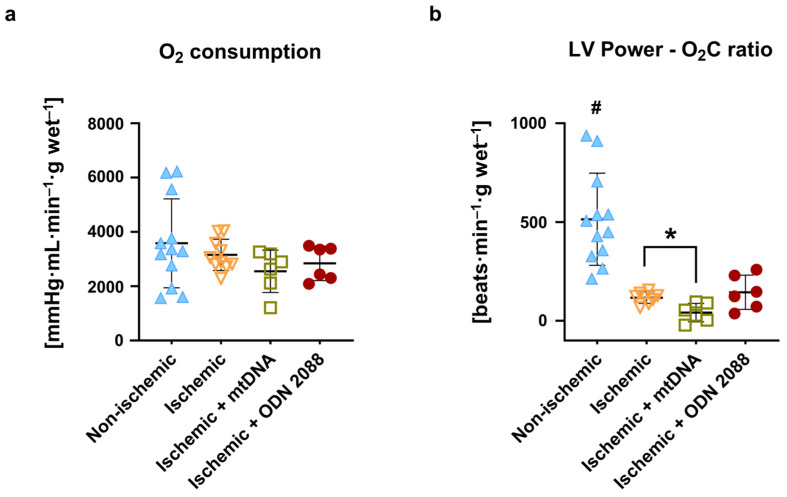
Post-ischemic cardiac oxygen metabolism at 60 min reperfusion. (**a**) Oxygen consumption; (**b**) LV power–O_2_C ratio as a measurement of oxygen efficiency. LV power: left ventricular power (heart rate–developed pressure–cardiac output product); O_2_C: oxygen consumption; min: minutes; ^#^ *p* < 0.05 vs. all ischemic groups; * *p* < 0.05 between indicated groups; *n* = 6 to 12 per group.

At the end of reperfusion, oxygen consumption was comparable among all groups (Figure 3a), whereas oxygen efficiency, expressed as LV power–oxygen consumption ratio (Figure 3b), was significantly different between non-ischemic vs. all ischemic groups (*p* < 0.05 for all). Treatment of ischemic hearts with mtDNA at the beginning of reperfusion significantly reduced oxygen efficiency compared to untreated ischemic hearts (*p* < 0.05), indicating an impairment in the use of oxygen to produce energy.

### 2.5. Mitochondrial Integrity During Reperfusion

**Figure 4:**
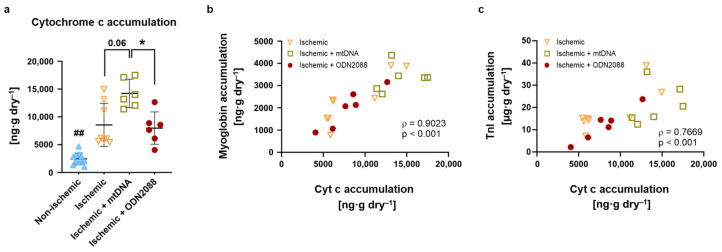
Mitochondrial integrity during reperfusion. (**a**) Cytochrome c accumulation over 60 min reperfusion in perfusate. (**b**) Correlation of myoglobin with Cyt C, release over 60 min reperfusion for both, in ischemic groups only (non-ischemic hearts not included). (**c**) Correlation of TnI with Cyt C, release over 60 min reperfusion for both, in ischemic groups only (non-ischemic hearts not included). Cyt C: cytochrome c; TnI: troponin I; ^##^ *p* < 0.01 vs. all ischemic groups; * *p* < 0.05 between indicated groups; *n* = 6 to 12 per group (**a**) and *n* = 20 x − y pairs (**b**,**c**).

Cytochrome c, a marker of mitochondrial integrity, was assessed in perfusate collected at the onset and end of reperfusion, and its accumulation during reperfusion was calculated. Accumulation of cytochrome c (Figure 4a) was significantly increased in all ischemic groups compared to non-ischemic hearts (*p* < 0.01). Treatment of ischemic hearts with mtDNA appeared to increase cytochrome c accumulation in relation to ischemic hearts (*p* = 0.06), and cytochrome c accumulation was significantly elevated in hearts treated mtDNA versus those treated with ODN2088 (*p* < 0.05). Among hearts that underwent ischemia, cytochrome c accumulation positively correlated with accumulation of both myoglobin (Figure 4b) and TnI (Figure 4c; *p* < 0.001 for both).

### 2.6. Oxidative Stress in Cardiac Tissue at the End of Perfusion

**Figure 5:**
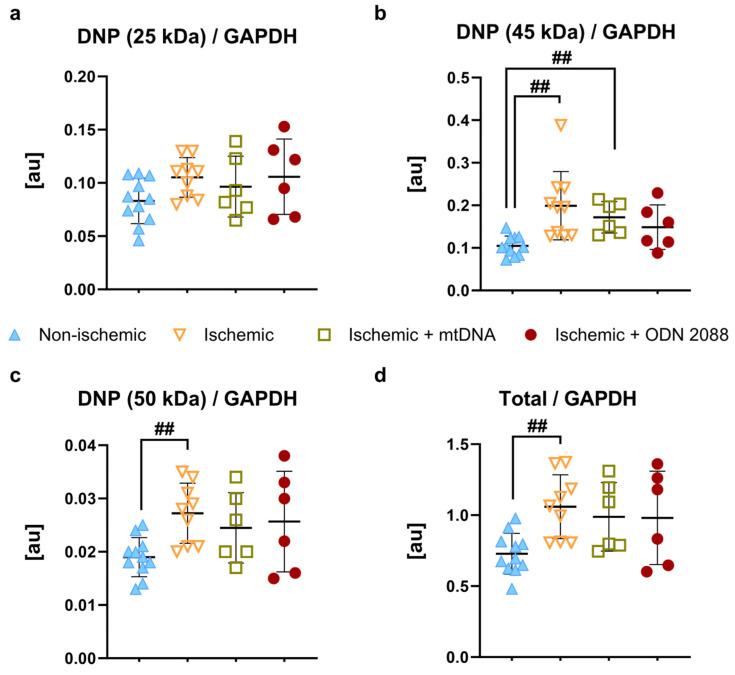
Oxidative stress in cardiac tissue at 60 min of reperfusion. Quantification of protein carbonylation bands at (**a**) 25 kDa, (**b**) 45 kDa, (**c**) 50 kDa and (**d**) total weighted sum of bands normalized to GAPDH protein content in cardiac tissue harvested at the end of reperfusion. au: arbitrary units; DNP: 1–3 dinitrophenylhydrazone moieties; GAPDH: glyceraldehyde 3-phosphate dehydrogenase; ^##^ *p* < 0.01 between indicated groups; *n* = 6 to 11 per group.

Oxidative stress in cardiac tissue was assessed through immunoblot detection of carbonyl groups introduced into proteins. As shown in Figure 5, protein carbonylation was increased by ischemia (band 45 kDa: *p* < 0.01 compared to non-ischemic hearts; band 50 kDa: *p* < 0.01 compared to non-ischemic hearts; and total sum of analyzed bands: *p* < 0.01 compared to non-ischemic hearts). For one band (45 kDa), protein carbonylation in mtDNA-treated hearts was similar to ischemic hearts, significantly higher than non-ischemic hearts (*p* < 0.01).

### 2.7. Investigation of TLR9 Activation-Induced Downstream Cascade in Cardiac Tissue

**Figure 6:**
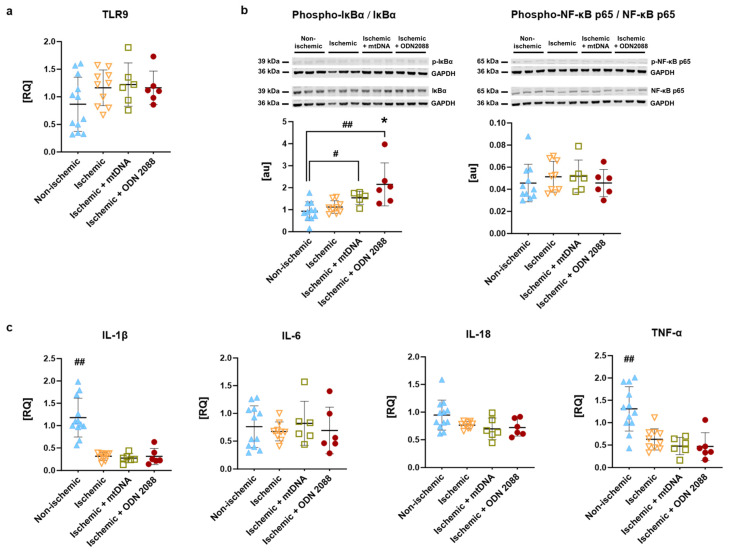
Investigation of TLR9-induced downstream cascade at 60 min of reperfusion. (**a**) mRNA expression of TLR9, (**b**) representative Western blots for phosphorylated and total proteins as well as the corresponding loading control (GAPDH) in top panels and phosphorylation-total ratios of IκBα and NF-κB p65, and (**c**) mRNA expression of IL-1β, IL-6, IL-18 and TNF-α. au, arbitrary units; IκBα, nuclear factor of kappa light polypeptide gene enhancer in B-cells inhibitor; IL: interleukin; NF-κB: nuclear factor kappa-light-chain-enhancer of activated B cells; RQ: relative quantity; TLR9: toll-like receptor 9; TNF-α: tumor necrosis factor α. ^#^ *p* < 0.05 between indicated groups; ^##^ *p* < 0.01 vs. all ischemic or between indicated groups; * *p* < 0.05 vs. ischemic; *n* = 6 to 12 per group.

In cardiac tissue procured at end of 60 min reperfusion, mRNA expression of TLR9 and downstream targets, as well as activation of nuclear factor kappa-light-chain-enhancer of activated b cells (NF-κB), are presented in Figure 6. As shown in Figure 6a, mRNA expression of TLR9 demonstrated a tendency towards higher expression in ischemic compared non-ischemic hearts. Activation of NF-κB, the main downstream target of TLR9 activation, was investigated through phosphorylation of its upstream activator, nuclear factor of kappa light polypeptide gene enhancer in B-cells inhibitor, alpha (IκBα), and of NF-κB p65 (Figure 6b). Ischemic hearts treated with mtDNA and ODN2088 at the beginning of reperfusion demonstrated significantly increased phosphorylation of IκBα compared with non-ischemic hearts (*p* < 0.05 vs. mtDNA treatment; *p* < 0.01 vs. ODN2088 treatment). Treatment of ischemic hearts with ODN2088 significantly increased IκBα phosphorylation compared to ischemia alone (*p* < 0.05). However, phosphorylation of IκBα in the mtDNA- and ODN2088-treated hearts was not associated with greater phosphorylation of the NF-κB p65 subunit. Finally, mRNA expression for targets downstream of NF-κB activation, interleukin-1beta (IL-1β), interleukin-6 (IL-6), interleukin-18 (IL-18) and TNF-α were determined in cardiac tissue (Figure 6c). Whereas mRNA expression of cardiac IL-6 and IL-18 was comparable among groups, mRNA expression of cardiac IL-1β and TNF-α was significantly lower in all ischemic conditions compared to the non-ischemic group (*p* < 0.01).

## 3. Discussion

We have investigated the effects of mtDNA in cardiac post-ischemic recovery using a pre-clinical model of heart donation after circulatory death. To do so, we performed intracoronary delivery of mtDNA (TLR9-activator) or a TLR9-antagonist (ODN2088) to DCD hearts at the beginning of reperfusion after a period of global, normothermic ischemia. We demonstrated that addition of exogenous mtDNA reduced cardiac recovery, aggravating post-ischemic contractile and metabolic dysfunction. Metabolic dysfunction was determined by a reduction in oxygen efficiency (the amount of work performed per oxygen consumed) compared to untreated, ischemic hearts, which was likely caused by disrupted mitochondrial integrity, and is consistent with the observed trend for greater release of cytochrome c. Furthermore, infusion of ODN2088 (TLR9 antagonist) did not modify post-ischemic cardiac contractile or metabolic recovery, but promoted phosphorylation of IκBα (activator of NF-kB) and appeared to decrease cell death. As DCD cardiac grafts are exposed to damaging conditions, including a period of warm ischemia, prior to procurement, mitochondrial integrity may be damaged, corresponding to a release of mtDAMPs, as observed with cytochrome c release upon reperfusion. As cardiac DCD grafts are often preserved with machine perfusion, hearts will be exposed to mtDAMPs present in the donor blood and/or released ex situ from cardiac mitochondria. Given the negative effects of circulating mtDNA on cardiac functional and metabolic recovery, circulating mtDAMPs, and particularly mtDNA, are of clinical relevance as targets for therapies aimed at optimizing graft quality and post-transplant outcomes.

Although little is known about the effects of mtDNA on post-ischemic recovery of DCD cardiac grafts, a few studies have been executed in animal models of warm cardiac ischemia. In a rat model of myocardial infarction, purified mtDNA, intravenously administered prior to 30 min regional ischemia and 24 h reperfusion, increased infarct size compared to ischemia alone [22]. Furthermore, this mtDNA treatment provoked significant augmentations in the protein content of TLR9 and caspase-3 (protein involved in apoptosis) in cardiac tissue harvested at the end of reperfusion. These effects of mtDNA on infarct size and tissue protein content were blocked when chloroquine, a TLR9 inhibitor, was infused together with mtDNA [22]. Comparable results for mtDNA-induced aggravation of infarct size have been reported in a rat model of global ischemia in which hearts underwent 15 min of global ischemia and 2 h reperfusion with treatment of mtDNA at the beginning of ischemia [23]. However, in another murine study of myocardial infarction with 20 min regional ischemia followed by 60 min reperfusion, administration of mtDNA at the beginning of reperfusion did not exacerbate infarct size [24]. Taken together, reports in pre-clinical models suggest that administration of mtDNA prior to, or at the time of, warm ischemia increases infarct size, but that this effect is dependent on dosage and/or timing of administration, and that negative effects may involve a heightened TLR9-dependent inflammatory response. However, these studies did not evaluate effects of mtDNA on post-ischemic recovery of ventricular or metabolic function [22,23,24].

Reduced oxidative phosphorylation likely contributes to the decreased ventricular function that we observed following treatment with mtDNA. Indeed, we measured a reduction in the ratio of power generated by the left ventricle per oxygen consumed in ischemic hearts treated with mtDNA compared with untreated hearts, indicating a lower mitochondrial efficiency. Consistent with these results, we measured a tendency towards increased cytochrome c release in mtDNA-treated hearts compared with untreated ischemic hearts, indicating greater mitochondrial damage. These findings are supported by the report of a reactive oxygen species-induced shortening in mitochondrial depolarization time in isolated, adult murine cardiomyocytes treated with mtDNA for 24 h [25], as faster mitochondrial depolarization is a sign of mitochondrial collapse and reduced ATP generation. Interestingly, administration of oligodeoxynucleotides containing CpG motifs, much like mtDNA, triggered metabolic alterations that led to decreased levels of ATP in both healthy isolated mouse hearts and cardiomyocytes; however, corresponding contractile function was not reported [26]. Furthermore, it has been proposed that this decrease in ATP levels is caused by a TLR9-induced reduction in SERCA2 activity, which modulates Ca^2+^ handling between the sarcoplasmic/endoplasmic reticulum and mitochondria, leading to lower metabolic rates and mitochondrial ATP levels, as potentially protective, energy preserving mechanisms [18]. The reduced ventricular function that we observed fits with the concept of reduced ATP production. Thus, under the conditions investigated in our study, reduced ventricular function likely results from mtDNA-induced mitochondrial damage that leads to decreased mitochondrial integrity and oxygen efficiency.

Despite impaired post-ischemic cardiac functional and metabolic recovery with mtDNA treatment, we did not observe a statistically significant activation of TLR9 and innate inflammatory responses. Using in vitro models, it has been demonstrated that 24 h of incubation of cardiomyocytes with purified mtDNA leads to higher mRNA expression of pro-inflammatory cytokines (IL-1β and IL-6) and a greater content of phosphorylated NF-kB [27], all signs of a mtDNA-induced pro-inflammatory state in treated cells. Furthermore, Bliksøen and colleagues reported that extracellular mtDNA provoked cell death in a dose-dependent manner in murine adult cardiomyocytes after 17 h of incubation [25]. These authors also demonstrated that extracellular mtDNA is internalized by murine adult cardiomyocytes after 24 incubation and that extracellular mtDNA promotes NF-kB activation both in vitro and in vivo [25]. Compared to previous pre-clinical studies, the dose of mtDNA used in our study was relatively low [22,23,25,27]. The lack of activation of innate inflammatory responses reported in our study might be due to the lower dose and the relatively short period of reperfusion in comparison to previous pre-clinical studies in vitro and in vivo [22,23,25,27].

In our isolated heart model of DCD, ODN2088 (TLR9 antagonist) appeared to decrease cell death, without modifying ventricular function. Furthermore, our findings do not provide evidence for an ODN2088-induced reduction in TLR9 activity, but rather the opposite was observed, namely a significantly greater IκBα phosphorylation. Thus, the effects of ODN2088 may result from stimulation of PI3K/Akt and/or Erk signaling pathways, as reported in other models [20,21], and are in line with the recognized cardioprotective effects of these pathways upon cardiac reperfusion [28,29]. Genetic deletion of TLR9 has been described as cardioprotective following 35 min of global ischemia and 1 h reperfusion [30]. Specifically, total ablation of TLR9 improved post-ischemic cardiac contractile function, with a reduction in infarction area, extracellular release of creatine kinase (marker of cell death) and mRNA expression of pro-inflammatory markers (TNF-α, IL-1β, IL-6) [30]. Interestingly, effects of genetic TLR9 ablation were mimicked when wild type hearts were treated with DNase I as a pre-conditioning therapy [30]; circulating mtDNA levels were decreased, post-ischemic cardiac contractile function improved and cell death was reduced without modifying innate inflammation. Furthermore, one pre-clinical study in cardiac myocardial infarction revealed that pre-ischemic treatment of animals with a TLR9 agonist (ODN1826) was cardioprotective, whereas pre-ischemic treatment with a TLR9 antagonist (ODN2088) did not modify cardiac contractile recovery [31]. Whereas this study is not fully comparable to our study due to the differing model of cardiac ischemia and to the application of TLR9 modulators as pre-ischemic therapies, it suggests that use of TLR9 agonists confer cardioprotection, while TLR9 antagonists do not alter post-ischemic cardiac recovery. The latter results, although in the myocardial infarction setting, agree with the lack of effect on post-ischemic contractile and metabolic function reported in our study. However, controversy persists regarding the impact of using TLR9 agonists in the context of cardiac ischemia and reperfusion, as differing results have been linked with the use of ODN1826 (TLR9 agonist) as cardioprotective [31] or as promoter of cardiac damage [32]. Thus, our findings, along with several pre-clinical studies, support a role for TLR9 inhibition in cardioprotective therapies; however, additional investigations are required to identify the precise mechanisms, as well as to determine the efficacy when therapies are administered only at the time of reperfusion (not earlier), such as the clinical settings of acute myocardial infarction and DCD heart transplantation.

### Limitations

We chose to perform these experiments entirely in an ex-situ perfusion system to precisely control the experimental conditions and limit the variability associated with the conditions surrounding circulatory arrest, such as hemodynamic instability and alterations in circulating factors and metabolites prior to warm ischemia. In clinical DCD, hearts would be exposed to variable conditions after the withdrawal of life-sustaining therapy, such as a hyperdynamic phase or a catecholamine surge prior to ischemia [33,34,35]. Furthermore, no echocardiographic analyses were performed; however, these could be an interesting addition to provide a more complete ventricular function assessment. Additionally, histological analysis of cardiac tissue at the end of reperfusion is of great mechanistic interest and should be performed in further studies to provide additional information about the effects of mtDNA at the tissue level. To ascertain the holistic mechanism of action of circulating mtDNA in cardiac tissue, further studies to assess parameters of mitochondrial function (such as, mitochondrial respiration and ATP generation, mitochondrial production of ROS and opening of mitochondrial permeability transition pore) would provide additional information in the mtDNA-induced downstream pathways and may reveal novel cardioprotective targets. Also, studies in large animals are needed to better understand the role of mtDNA in DCD heart donation.

Concerning the mechanism of action of mtDNA, it is important to mention that we centered our study on the investigation of mtDNA-mediated TLR9 activation pathway, due to the emerging role of TLR9 modulation as cardioprotective therapy. However, additional investigations into other mtDNA-mediated innate inflammation pathways, such as NLRP3 inflammasome and cGAS-STING DNA-sensing, are also important to consider and may provide insights into alternative cardioprotective therapies.

## 4. Materials and Methods

### 4.1. Animals and Ethics Statement

Male Wistar rats (Janvier Labs; Le Genest-Saint-Isle, France) were housed under standard conditions and provided unrestricted access to food and water.

The experimental protocols were executed in accordance with ARRIVE guidelines and guidelines of the European Convention for Animal Care. Protocols received approval from both the Swiss animal welfare authorities and the Ethics Committee for Animal Experimentation in Bern, Switzerland. Surgical procedures were conducted under general anesthesia, and the utmost care was taken to minimize stress experienced by the animals.

### 4.2. Experimental Design

**Figure 7:**
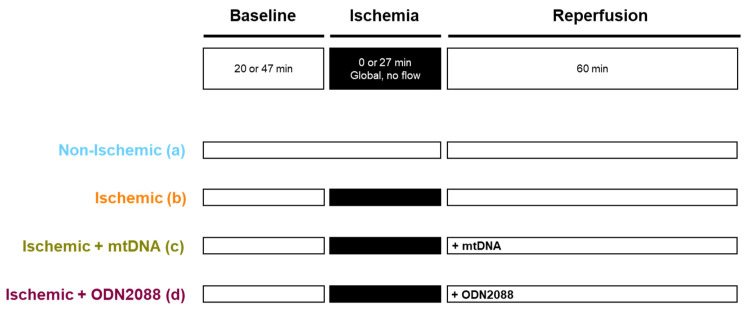
Experimental design. Hearts were randomly allocated to 1 of 4 parallel, experimental arms. All hearts initially underwent 20 min, aerobic baseline with left ventricular loading and Krebs–Henseleit buffer supplemented with 0.5 mM lactate, 1.2 mmol/L palmitate and 1.5% bovine serum albumin (see details in Section 4). While the non-ischemic group (group a) did not undergo any warm ischemia, the remaining three groups were submitted to 27 min global, warm ischemia. Subsequently, all hearts were reperfused for 60 min using Krebs–Henseleit buffer without supplementation. Reperfusion was initiated in unloaded mode for the first 10 min and then switched to loaded mode for the remaining 50 min. At the beginning of reperfusion, ischemic groups received either no additional agent (group b), 67 µg of purified mtDNA in perfusion buffer (group c), or 60 µg of ODN2088 in perfusion buffer (group d). mtDNA: mitochondrial DNA.

Our established isolated heart model was used for this study [36,37], with minor modifications. Four parallel study arms were included (see Figure 7): a non-ischemic group (*n* = 12) that underwent extended aerobic baseline perfusion instead of global, warm ischemia, and three ischemic groups. The ischemic groups were subjected to 27 min global, warm ischemia followed by differing conditions at the onset of reperfusion as follows: (i) standard reperfusion conditions (no treatment at reperfusion onset); (*n* = 10) or, (ii) addition of mtDNA isolated from rat liver into circulating perfusate (final concentration of 479 ng/mL) (*n* = 6), or (iii) addition of TLR9 antagonist ODN2088 (InvivoGen; San Diego, CA, USA) into circulating perfusate (final concentration 429 ng/mL; *n* = 6). The dose of mtDNA used in this study was greater than circulating levels observed in human DCD donors during transplantation of kidney, liver and lung, but not heart (approximately 8 ng/mL prior to organ procurement) [14], as it is expected that extracellular mtDNA levels will be increased during reperfusion, due to reperfusion-induction of mitochondrial damage [38]. However, compared to previous pre-clinical studies, the dose of mtDNA used in our study was relatively low [22,23,25,27]. Concerning ODN2088, the lowest dose recommended by the vendor was used to evaluate the effects of a dose considered safe for translation into clinical practice. Both agents were administered at the beginning of reperfusion and were dissolved in the standard reperfusion buffer (see isolated heart preparation and perfusion for details). The duration of 27 min of warm, global ischemia has been chosen based on previous laboratory studies [36,39,40,41,42,43], as it has been shown to be a reproducible and appropriate balance between post-ischemic injury and cardiac recovery.

### 4.3. Isolated Heart Preparation and Perfusion

Young adult male rats (12 weeks of age) were anesthetized by an intraperitoneal injection of 100 mg/kg of ketamine (Vetoquinol AG; Bern, Switzerland) and 10 mg/kg of xylazine (Vetoquinol AG; Bern, Switzerland). During anesthesia, blood oxygen levels were constantly monitored using a toe-clip pulse oximeter (ADInstruments; Dunedin, New Zealand) and oxygen was supplied when blood oxygen saturation dropped below 95%. After loss of the pedal reflex, hearts were explanted and cannulated on an isolated perfusion system. Time elapsed between diaphragm transection and initial retrograde perfusion of heart in Langendorff mode was no longer than 150 s.

On the perfusion system, hearts underwent 20 min of working (left ventricle loaded) baseline perfusion with a modified Krebs–Henseleit buffer containing 118 mM NaCl, 4.7 mM KCl, 1.2 mM KH_2_PO_4_, 1.25 mM CaCl_2_·2H_2_O, 1.2 mM MgSO_4_·7H_2_O, 25 mM NaHCO_3_, and 11 mM glucose, supplemented with 1.2 mM palmitate, 1.5% bovine serum albumin (BSA) and 0.5 mM lactate. Baseline perfusion was followed by 27 min of global warm (37 °C) ischemia. Reperfusion was initiated for 10 min in an unloaded mode, followed by 50 min with left ventricular loading using the same modified Krebs–Henseleit buffer as mentioned above, but without the addition of palmitate, BSA and lactate. Reperfusion afterload pressure (aortic line) was maintained at 60 mmHg to ensure sufficient coronary perfusion.

Detailed methods for the isolation of liver mtDNA, functional and biochemical assessment, measurements of markers of cell death, mitochondrial damage and oxidative stress, as well as Western blot analysis and measurements of mRNA expression levels are described in the Appendix A.

### 4.4. Statistical Analysis

Statistical analyses were performed with GraphPad Prism 8 (GraphPad Software, Inc.; La Jolla, CA, USA). Outliers were detected with Tukey’s box and whiskers plot and removed from the statistical analyses [44,45]. Spearman rank order correlation was used to investigate associations between the accumulation of cytochrome c and markers of cell death in the perfusate during reperfusion. For an overview of the differences between the experimental groups, the Kruskal–Wallis test was used. When overall results were statistically significant, pairwise comparisons were evaluated with Mann–Whitney U tests. All *p*-values were two-tailed and adjusted for multiple comparisons using a modified, sequential rejective Bonferroni procedure [46]. *p*-values are reported after correction and considered statistically significant if <0.05. All values are expressed as mean ± standard deviation unless stated otherwise.

## 5. Conclusions

To conclude, we report that administration of exogenous mtDNA to DCD hearts upon reperfusion reduces recovery of cardiac contractile and metabolic function. Our oxygen efficiency and cytochrome c results suggest that loss of mitochondrial integrity and mitochondrial dysfunction are mechanisms by which mtDNA exerts its detrimental effects. These findings provide new insight into the role of mtDNA in the post-ischemic heart, by evaluating acute effects that may occur earlier and, in addition to, previously reported TLR9-dependent changes. ODN2088, a TLR9 antagonist, appears to decrease cell death and trigger earlier steps in innate inflammation without modifying cardiac recovery, potentially via alternative signaling pathways. Further investigation is needed to better understand the roles of mtDNA and TLR9 in cardiac DCD donation. Furthermore, investigation of different strategies that block or prevent effects of extracellular mtDNA, such as the use of DNase I, are promising as new therapeutic areas for consideration in DCD. Additionally, the combination of DNase I and mitochondrial-targeted endonuclease III [23], to maintain the mitochondrial integrity in ischemic cardiomyocytes, would also be worthwhile testing in a DCD setting.

## Figures and Tables

**Table 1:** Baseline characteristics in isolated hearts after 20 min aerobic perfusion. Values are shown for the four experimental groups. CF: coronary flow; CO: cardiac output; LV power: left-ventricular power (heart rate–developed pressure–cardiac output product); mtDNA: mitochondrial DNA; ODN2088: TLR9 antagonist. Values are expressed as means ± SD. ^§§^ *p* < 0.01 vs. ischemic + ODN2088.

	Non-ischemic (*n* = 12)	Ischemic (*n* = 10)	Ischemic + mtDNA (*n* = 6)	Ischemic + ODN2088 (*n* = 6)
LV power (mmHg·beats·mL·min^−2^)	2.58 × 10^6^ ± 4.47 × 10^5^	2.57 × 10^6^ ± 5.34 × 10^5^	2.66 × 10^6^ ± 4.10 × 10^5^	2.18 × 10^6^ ± 4.42 × 10^5^
CO (mL·min^−1^)	77.5 ± 9.0	73.8 ± 9.7	77.3 ± 6.5	67.9 ± 11.2
CF (mL·min^−1^)	34.3 ± 4.5	33.6 ± 5.3	33.3 ± 3.3	31.3 ± 5.2
O_2_ consumption (mmHg·mL·min^−1^·g wet^−1^)	5799 ± 1208	6153 ± 1135	6412 ± 421 ^§§^	4808 ± 1041
LV power–oxygen consumption ratio (beats·min^−1^·g wet)	457 ± 100	418 ± 69	415 ± 66	456 ± 36
Body weight (g)	398 ± 22	397 ± 18	413 ± 17	397 ± 15
Heart weight (g)	1.9 ± 0.3	1.8 ± 0.1	1.7 ± 0.2	2.1 ± 0.4
Body–heart weight ratio	209 ± 30	221 ± 14	240 ± 22	198 ± 36

## Data Availability

The original contributions presented in this study are included in the article. Further inquiries can be directed to the corresponding author.

## Abbreviations

The following abbreviations are used in this manuscript:

CF	Coronary flow
CO	Cardiac output
Cyt c	Cytochrome c
DAMPs	Damage-associated molecular patterns
DBD	Donation after brain death
DCD	Donation after circulatory death
IFN-β	Interferon beta
IL-1β	Interleukin 1 beta
IL-6	Interleukin 6
IL-18	Interleukin 18
IR	Ischemia–reperfusion
IRF7	Interferon regulatory factor 7
IκBα	Nuclear factor of kappa-light chain-enhancer of activated b cells inhibitor alpha
LV power	Left ventricular power
mtDAMPs	Mitochondrial damage-associated molecular patterns
mtDNA	Mitochondrial DNA
NF-κB	Nuclear factor kappa-light chain-enhancer of activated b cells
ODN2088	Toll-like receptor 9 antagonist
PI3K	Phosphoinositide 3 kinase
TLR9	Toll-like receptor 9
TNF-α	Tumor necrosis factor alpha
TnI	Cardiac troponin I

## Appendix A

### Appendix A.1. Isolation of Liver mtDNA

Firstly, mitochondria were extracted from livers of male Wistar rats using standard differential centrifugation. All steps were carried out in a temperature range of 4–8 °C as previously described [47]. Briefly, rat livers were explanted, immersed in isolation buffer (pH 7.2–7.3) containing 250 mM sucrose, 1 mM EGTA, 20 mM Tris-HCl and 0.1% BSA, and cut into small pieces. Tissue was homogenized in a Potter–Elvehjem homogenizer (B. Braun; Melsungen, Germany) at 700 rpm. Consecutive centrifugation steps from 800× *g* to 8000× *g* were performed to isolate mitochondria. The final pellet containing mitochondria was resuspended in isolation buffer without BSA and stored at −80 °C.

Secondly, mitochondria isolated as described above were used for the extraction of mtDNA with a QIAmp^®^ DNA Blood Midi Kit (Qiagen; Hilden, Germany), following the manufacturer’s guidelines. Purified mtDNA from several livers was pooled and sonicated with the Ultrasonic Homogenizer Series HD 2000.2 (Bandelin electronic GmbH; Berlin, Germany). DNA was quantified with the NanoDrop 2000 (Thermo Fisher Scientific; Waltham, MA, USA), aliquoted, and stored at −20 °C until use. Prior to use in isolated heart experiments, the mtDNA was characterized as follows: Purity of mtDNA was assessed by two means: (i) assessment of contamination with nuclear genes; and (ii) assessment of contamination with other mtDAMPs. Contamination of mtDNA with nuclear genes was investigated by real-time PCR amplification of three mitochondrial genes (NADH dehydrogenase 1: ND1; cytochrome b: CYTB; and 16S RNA: RNR2) and three nuclear genes (Beta-2-Microglobulin: B2M; NADH:Ubiquinone Oxidoreductase Core Subunit V1: Ndufv1; b-actin: ACTB). Primer sequences are reported in Table A1. DNA extracted from cardiac tissue was used as positive control for both types of genes. Whereas all mitochondrial genes were detected in isolated mtDNA at similar levels to those found in DNA extracted from cardiac tissue, only the nuclear gene Beta-2-Microglobulin was detected in the isolated mtDNA and its presence was negligible compared with mitochondrial genes. Furthermore, contamination of mtDNA with other mtDAMPs was investigated by quantification of cytochrome c according to established protocols in the lab [39]. Presence of cytochrome c in isolated mtDNA was also considered as negligible.

**Table A1:** Sequences of primers used in liver mitochondrial DNA characterization. ACTB: b-actin; B2M: Beta-2-Microglobulin; CYTB: cytochrome b; ND1: NADH dehydrogenase 1; NDUFV1: Ubiquinone Oxidoreductase Core Subunit V; RNR2: 16S RN.

Target	Forward Primer Sequence	Reverse Primer Sequence
ND1	5′-CGGCTCCTTCTCCCTACAAA-3′	5′-AATGGTCCTGCGGCGTATT-3′
CYTB	5′-ACAGGATTAAACTCCGACGCA-3′	5′-GTTTGTTGGGAATGGAGCGT-3′
RNR2	5′-GAAGAGGCTGGAATCTCCCAA-3′	5′-CTAGGGTAACTTGGTCCGTTGA-3′
B2M	5′-GAGGAAACTGAGGGGAGTAGG-3′	5′-AGACCCCAGCCTTTACACAAT-3′
NDUFV1	5′-GCACAGCTGCGGTTATTGTTA-3′	5′-ATCCCCCTTCACAAATCGGG-3′
ACTB	5′-CTAAGGCCAACCGTGAAAAG-3′	5′-CATCACAATGCCAGTGGTACG-3′

### Appendix A.2. Functional and Biochemical Assessment

A micro-tip pressure catheter (Millar; Houston, TX, USA) was inserted into the left ventricle via the left atrial cannula to continuously record ventricular pressure, which allows determination of several parameters of ventricular function, such as developed pressure (DP) and heart rate. Coronary flow and cardiac output were monitored using flowmeters (Transonic Systems Inc.; Ithaca, NY, USA) located in both preload and afterload lines. All collected data was recorded using the PowerLab data acquisition system (ADInstruments; Dunedin, New Zealand).

Circulating perfusate and coronary effluent samples were collected at multiple time points. Blood gas analysis with a COBAS b 123 blood gas analyzer (Roche; Basel, Switzerland) was used to measure pO_2_ in perfusate samples, for the determination of cardiac oxygen consumption. Perfusate samples were then stored at −80 °C for later use.

Cardiac tissue was snap-frozen in liquid nitrogen at the end of the perfusion protocol and stored at −80 °C for further analysis of oxidative stress, Western blot and mRNA expression.

### Appendix A.3. Markers of Cell Death, Mitochondrial Damage and Oxidative Stress

Perfusate samples were used for the determination of markers of cell death using the commercially available ELISA kits for rat myoglobin and troponin I (Life Diagnostics; West Chester, PA, USA).

To assess mitochondrial damage, the release of cytochrome c was quantified using the Rat/Mouse Cytochrome c Quantikine ELISA kit (R&D Systems; Minneapolis, MN, USA).

Oxidative stress was determined as protein carbonylation in whole heart tissue samples using the OxyBlot™ Protein Oxidation Detection kit (Merck Millipore; Burlington, MA, USA), as previously described [39].

### Appendix A.4. Western Blot Analysis

Protein samples were obtained by mechanically disrupting frozen tissue using a mortar and pestle, followed by homogenization in a customized RIPA lysis buffer using the TissueLyser II (Qiagen; Hilden, Germany). Proteins were subsequently separated using SDS-PAGE with Criterion™ TGX™ precast gels (Bio-Rad; Hercules, CA, USA) and transferred onto nitrocellulose membranes (LI-COR Biosciences; Bad Homburg, Germany). Western blots were performed using rabbit monoclonal primary antibodies against phosho-NF-κB p65 (Ser 536; #3033), NF-κB p65 (#8242), phospho-IκBα (Ser32; #2859) and mouse monoclonal anti-IκBα (#4814; all from Cell Signaling, Beverly, MA, USA). Mouse monoclonal anti-GAPDH (sc-365062) was obtained from Santa Cruz Biotechnology (Heidelberg, Germany) and used to detect GAPDH (loading control). Two fluorescent secondary antibodies were used: (i) goat anti-rabbit (Invitrogen; Carlsbad, CA, USA), and (ii) goat anti-mouse (LI-COR Biosciences; Lincoln, NE, USA).

Immunodetection of Western blots was performed with a near-infrared imaging system (Odyssey, LI-COR Biosciences; Bad Homburg, Germany).

### Appendix A.5. mRNA Expression Levels

Total cardiac RNA was isolated using the miRNeasy kit (Qiagen; Hilden, Germany) following the provided guidelines. Quality and quantity of RNA were assessed using Nanodrop 2000/2000c optical density measurements (Thermo Fisher Scientific; Waltham, MA, USA). Oligo-dT first strand cDNA synthesis was performed using 1 μg RNA, and subsequently, 10 ng cDNA was used for real-time PCR amplification utilizing the SYBR Green method (Quantitect SYBR Green; Qiagen, Hilden, Germany) in a ViiA 7 PCR System (Applied Biosystems; Foster City, CA, USA). The relative quantities of target genes (TLR9, IL-1β, IL-6, IL-18 and TNF-α) were computed using the Design & Analysis Software Version 2.6.2 (Thermo Fisher Scientific; Waltham, MA, USA) after normalization by two reference genes (RPLP0 and Ywhaz). Stability of reference genes was confirmed via RefFinder [48]. Primer sequences can be found in Table A2. Negative controls without template (NTCs) were included for each primer pair during the qPCR stage. To control for genomic DNA contamination, a reaction lacking reverse transcriptase (RT) was included for each sample and primer pair during the reverse transcription step. Moreover, NTCs were included for each primer pair during the RT step.

**Table A2:** Sequences of primers used in determination of cardiac mRNA expression. IL-1β: interleukin-1beta; IL-18: interleukin-18; IL-6: interleukin-6; TLR9: toll-like receptor 9; TNF-α: tumor necrosis factor α.

Target	Forward Primer Sequence	Reverse Primer Sequence
TLR9	5′-TCCATTTTCCATCATGGTTCTCTG-3′	5′-GAGGCTTCAGTTCACAGGGT-3′
IL-6	5′-CCAGTTGCCTTCTTGGGACT-3′	5′-CTGGTCTGTTGTGGGTGGTA-3′
IL-18	5′-GACAAAAGAAACCCGCCTG-3′	5′-ACATCCTTCCATCCTTCACAG-3′
TNF-α	5′-ATGGGCTCCCTCTCATCAGT -3′	5′-AAATGGCAAATCGGCTGACG-3′
IL-1β	5′-AGGCTGACAGACCCCAAAAG-3′	5′-GGTCGTCATCATCCCACGAG-3′
